# Comparative genetic analysis of the novel coronavirus (2019-nCoV/SARS-CoV-2) receptor ACE2 in different populations

**DOI:** 10.1038/s41421-020-0147-1

**Published:** 2020-02-24

**Authors:** Yanan Cao, Lin Li, Zhimin Feng, Shengqing Wan, Peide Huang, Xiaohui Sun, Fang Wen, Xuanlin Huang, Guang Ning, Weiqing Wang

**Affiliations:** National Clinical Research Centre for Metabolic Diseases, State Key Laboratory of Medical Genomics, Shanghai Clinical Center for Endocrine and Metabolic Diseases, Shanghai Institute for Endocrine and Metabolic Diseases, Ruijin Hospital, Shanghai Jiaotong University School of Medicine, 200025 Shanghai, China

**Keywords:** Bioinformatics, Genomic analysis

Dear Editor,

The *ACE2* gene encodes the angiotensin-converting enzyme-2, which has been proved to be the receptor for both the SARS-coronavirus (SARS-CoV) and the human respiratory coronavirus NL63. Recent studies and analyses indicate that ACE2 could be the host receptor for the novel coronavirus 2019-nCoV/SARS-CoV-2^1,2^. Previous studies demonstrated the positive correlation of ACE2 expression and the infection of SARS-CoV in vitro^3,4^. A number of ACE2 variants could reduce the association between ACE2 and S-protein in SARS-CoV or NL63^5^. Therefore, the expression level and expression pattern of human ACE2 in different tissues might be critical for the susceptibility, symptoms, and outcome of 2019-nCoV/SARS-CoV-2 infection. A recent single-cell RNA-sequencing (RNA-seq) analysis indicated that Asian males may have higher expression of ACE2^6^. Currently, the clinical reports of 2019-nCoV/SARS-CoV-2 infection from non-Asian populations for comparison are very limited. A study from Munich reported four German cases, all of which showed mild clinical symptoms without severe illness^7^. However, the genetic basis of ACE2 expression and function in different populations is still largely unknown. Therefore, genetic analysis of expression quantitative trait loci (eQTLs)^8^ and potential functional coding variants in *ACE2* among populations are required for further epidemiological investigations of 2019-nCoV/SARS-CoV-2 spreading in East Asian (EAS) and other populations.

To systematically investigate the candidate functional coding variants in *ACE2* and the allele frequency (AF) differences between populations, we analyzed all the 1700 variants (Supplementary Table S1) in *ACE2* gene region from the ChinaMAP (China Metabolic Analytics Project, under reviewing) and 1KGP (1000 Genomes Project)^9^ databases. The AFs of 62 variants located in the coding regions of *ACE2* in ChinaMAP, 1KGP, and other large-scale genome databases were summarized (Supplementary Table S2). All of the 32 variants potentially affecting the amino acid sequence of *ACE2* in databases were shown (Fig. 1a). Previous study showed that the residues near lysine 31, and tyrosine 41, 82–84, and 353–357 in human ACE2 were important for the binding of S-protein in coronavirus^5^. The mutations in these residues were not found in different populations in our study. Only a singleton truncating variant of ACE2 (Gln300X) was identified in the ChinaMAP (Fig. 1a). These data suggested that there was a lack of natural resistant mutations for coronavirus S-protein binding in populations. The effects of low-frequency missense variants in populations for S-protein binding could be further investigated. The distributions of seven hotspot variants (Lys26Arg, Ile468Val, Ala627Val, Asn638Ser, Ser692Pro, Asn720Asp, and Leu731Ile/Leu731Phe) in different populations were shown (Fig. 1b). Six low-frequency loci (rs200180615, rs140473595, rs199951323, rs147311723, rs149039346, and rs73635825) were found to be specific in 1KGP database, the AFs of which were also low in the gnomAD and TopMed^10^ database. Only two of these six variants (rs200180615 and rs140473595) could be found in CHB (Han Chinese in Beijing) population with the AF < 0.01. Interestingly, the SNP rs2285666 with the highest AF in the 62 variants exhibited much higher AF in the ChinaMAP (0.556) and CHS (Han Chinese South, 0.557) populations compared to others (AMR, Ad Mixed American, 0.336; AFR, African, 0.2114; EUR, European, 0.235). In addition, the homozygous mutation rate in males (0.550) was much higher than females (0.310) in the Chinese population (Supplementary Table S2). Taken together, the differences in AFs of *ACE2* coding variants among different populations suggested that the diverse genetic basis might affect ACE2 functions among populations.

**Fig. 1:**
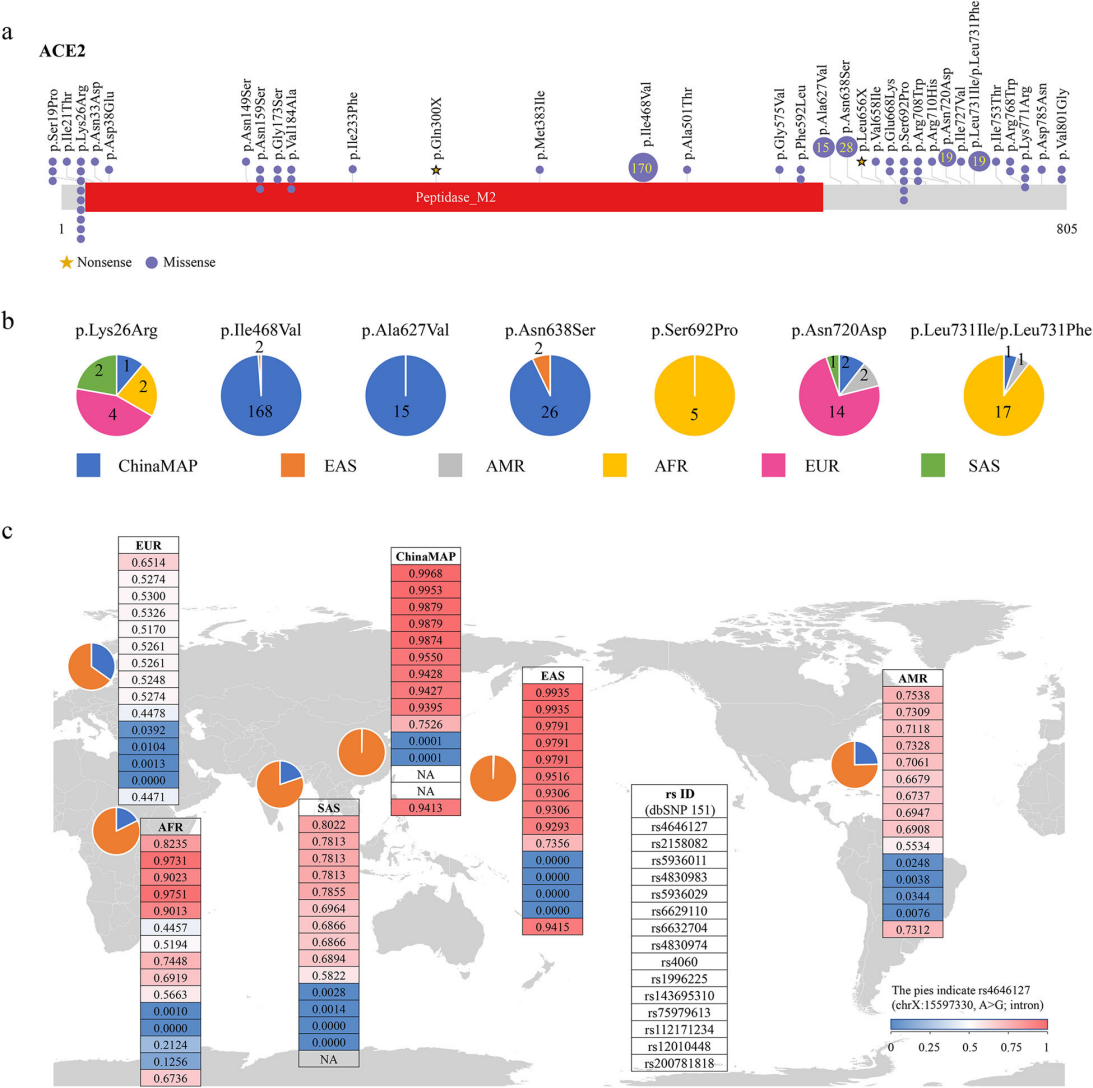
The coding-region variants and eQTL variants for *ACE2* in East Asian and other populations. **a** Schematics of 32 coding variants in *ACE2* identified in the ChinaMAP and 1KGP databases. Yellow stars indicate the nonsense variants; dots indicate the missense variants. The number of samples with hotspot variants was marked. **b** The distribution of hotspot missense mutations of ACE2 in different populations. The colors indicate different populations. **c** The distribution and the allele frequencies of representative eQTL variants for *ACE2* in different populations. Pie charts depict the allele frequencies of an intron variant of *ACE2* (rs4646127) in the world. Orange color denotes the frequency of alteration allele, and blue color denotes the reference allele. The allele frequencies of 15 eQTLs for *ACE2* gene are shown in tables. The color gradient from blue to red indicates the increasing of allele frequencies. The allele frequencies of INDEL variant rs200781818 were annotated by the gnomAD database. EAS, East Asian; EUR, European; AFR, African; SAS, South Asian; AMR, Ad Mixed American.

To analyze the distribution of eQTLs for *ACE2*, we used the Genotype Tissue Expression (GTEx) database (https://www.gtexportal.org/home/datasets). We found 15 unique eQTL variants (14 SNPs and 1 INDELs) for *ACE2* with *q* value ≤ 0.05 in 20 tissues from the GTEx database (rs112171234, rs12010448, rs143695310, rs1996225, rs200781818, rs2158082, rs4060, rs4646127, rs4830974, rs4830983, rs5936011, rs5936029, rs6629110, rs6632704, and rs75979613). The AFs of the 15 eQTL variants were compared among different populations. Notably, our results showed most of the 15 eQTL variants had much higher AFs in the ChinaMAP dataset and EAS populations compared to European populations (Fig. 1c and Supplementary Table S3). The AFs of the top 6 common variants (rs4646127, rs2158082, rs5936011, rs6629110, rs4830983, and rs5936029) were higher than 95% in EAS populations, whereas the AFs of these variants in European populations were much lower (52%–65%). All of the 11 common variants (AF > 0.05) and 1 rare variant (rs143695310) in the 15 eQTLs are associated with high expression of ACE2 in tissues (Supplementary Table S3). For instance, the eQTL variant rs4646127 (log allelic fold change = 0.314), which locates in the intron of *ACE2* gene, has the highest AFs in both of the ChinaMAP (0.997) and EAS (0.994) populations. Comparatively, the AFs of rs4646127 in EUR (0.651) and AMR (0.754) populations are much lower. These findings suggested the genotypes of *ACE2* gene polymorphism may be associated higher expression levels of ACE2 in EAS population.

Recent reports of the ACE2 expression analysis in lung tissues from Asian and Caucasian populations are still controversial. The single-cell RNA-seq analysis reported that the Asian donor had much higher ACE2 expression cell ratio than white and African-American donors^6^. In contrast, the ACE2 expression analysis using the RNA-seq and microarray datasets from control lung tissues indicated there were no significant differences between Asian and Caucasian, or male and female^11^. The ACE2-expressing cells are a very small part of cells in lung tissues^6^. The sample size and the purity of ACE2-positive cells in the selected samples would influence the conclusions. Our analysis showed the differences in distribution and AFs of eQTLs for ACE2 in different populations, indicating the diversity of ACE2 expression pattern in populations (Supplementary Table S3). Large-scale and multiple tissue-level analysis of single-cell RNA-seq would be more accurate for the expression analysis of ACE2 in different populations. In addition, our data showed the moderate difference in AFs of eQTLs between South Asian and EAS, which suggests the potential difference of ACE2 expression in different populations and ethnics in Asia (Fig. 1c).

In summary, we systematically analyzed coding-region variants in *ACE2* and the eQTL variants, which may affect the expression of ACE2 using the GTEx database to compare the genomic characteristics of *ACE2* among different populations. Our findings indicated that no direct evidence was identified genetically supporting the existence of coronavirus S-protein binding-resistant *ACE2* mutants in different populations (Fig. 1a). The data of variant distribution and AFs may contribute to the further investigations of ACE2, including its roles in acute lung injury and lung function^12^. The East Asian populations have much higher AFs in the eQTL variants associated with higher ACE2 expression in tissues (Fig. 1c), which may suggest different susceptibility or response to 2019-nCoV/SARS-CoV-2 from different populations under the similar conditions.

## Supplementary information

Supplementary Table S1

Supplementary Table S2

Supplementary Table S3

## Supplementary information

**Supplementary Information** accompanies the paper at (10.1038/s41421-020-0147-1).
